# Blind-Alley Hospital Employments

**Published:** 1913-10-04

**Authors:** 


### BLIND-ALLEY HOSPITAL EMPLOYMENTS.

### The Case of the Laboratory Boy.

At the present time when attempts are made to direct the employment of boys and girls who leave school into channels wherein these youthful workers will have scope for permanent effort, as opposed to occupations in which the temporary nature of the work is well recognised, it is worth while to pay some attention to the blind alleys that exist in hospital occupations. The chief of these is undoubtedly the temporary occupation which is given to boys in the laboratories in hospitals with medical schools attached, although somewhat similar blind alleys exist in other departments of hospital life.

The boy who is engaged to work in the hospital laboratories is usually taken from a fairly intelligent and active class. The work he has to do entails comparatively little expenditure of physical energy or strength, but demands a certain amount of knowledge, and in the course of time imparts a certain amount of additional knowledge which is valuable only when exploited in similar occupations. For instance, the boy attached to the bacteriological laboratory learns to prepare broths, culture media, and culture plates; he learns how to clean a microscope, to look after certain instruments, to handle and deal with animals used for injection and control experiments; he gets to know something about bacteriology and the elements, at least, of scientific work. Similarly, the boy in the physiological laboratory comes to know how to clean apparatus and set it up for experiments, he becomes acquainted with reagents and their uses. In the chemical laboratory similarly special knowledge is imparted to him; in the other laboratories there is an equal chance to learn. While he is labouring his hours are short; the work is by no means arduous; he is kindly and sympathetically treated; his wages are usually considerably above what he can earn in an office or as a van boy, and far in excess of what he would get were he apprenticed to a trade. In short, the prospect is alluring to a boy just leaving school, and there is usually no difficulty whatever in filling the vacancy. The applications are generally so numerous, where a vacancy is advertised, that the employers have an unlimited choice, and the selection is made from the best class.

### The Laboratory Boy's Future.

The pity of it is that such employment, so alluring to the boy, leads, in general, to a rut out of which it is exceedingly difficult to get when he grows older, and his economic worth increases. In other words there is little prospect for him. It is true those who object to this statement can point to laboratory boys who have risen to high eminence. Gull was a laboratory boy; we believe we are correct in stating that there are to-day certain teachers of the preliminary sciences in medical schools who, by dint of persevering effort, have raised themselves from the position of laboratory boys to the posts they now occupy. But these are exceptions which can be paralleled in every other blind-alley occupation. The fact remains that for the average laboratory boy, as for the average telegraph boy, there is no future in the department wherein he works for a couple of years. At the end of that time, when he gets too big for his work, he is dismissed or relegated to another department of hospital activity where his wages are certainly not in proportion to his economic value. The knowledge he has gained in the laboratories is, commercially, so much dunnage which serves no useful purpose since there is no cargo which it can support.

### A Suggested Reform.

This is putting the case for the laboratory boy baldly. It may be supplemented by stating that in many respects such work as he is called upon to perform in the laboratories unfits him for such work as he may afterwards be called upon to perform. All boys cannot hope to become permanently attached to laboratories or to remain in a scientific or pseudo-scientific occupation. These posts are eagerly competed for, and the supply by far exceeds the demand. A certain proportion of laboratory boys must, therefore, be thrown out of employment, unfitted to take up work which their age and their experience should readily bring them. Companions who have left school with them and have entered a trade surpass them in the race, and have a better economic prospect than is theirs. It is true, again, that in many cases, where they have shown their value and capacity for work, their employers take a sympathetic interest in their future, and are able to place them in a new occupation. But that is entirely a personal favour, and even if the boy proceeds to some other department of hospital work —and be it remembered that there are not so many departments open to him—he is handicapped in various ways.

The obvious way out of the difficulty seems to be to make the position of laboratory attendant a stepping-stone to higher and better paid posts, and to employ no boy who is not capable, with proper training, of filling these higher posts. With the increase of special departments in general hospitals such posts must increase in number, and it is far better that the men who fill them should be selected from youths who have already had hospital training, and who are, in some measure at least, imbued with what may be called the hospital spirit, than that they should be recruited from outsiders. Then, again, there are numerous posts in the hospital itself that may be filled by trained youths, although at present they are largely filled by men. Lift attendants, porters, gatekeepers, and gardeners are, in Continental hospitals, usually selected from among boys who have been working for the hospital in other departments, and have outgrown their usefulness for these departments. Abroad, too, the boy with hospital tastes has a chance of enlisting as a trained attendant or a male nurse. At present, with one exception, our Metropolitan hospitals do not cater for the demand for male nurses and attendants. A boy who wishes to take up this training, as an addition to his duties as valet or general man-servant, finds it exceedingly difficult to secure such training. There is no easy way of starting, for with the exception of this one hospital all the other institutions are closed to him. One other hospital which employs male nurses secures them from abroad, and does not undertake their training. A demand for such trained men is certainly arising, and it is proper that the hospitals should take it into consideration when they are in a position to do so.

Obviously a great deal depends on the boy himself. A boy with scientific tastes and some skill with his fingers, generally speaking, would do far better to get apprenticed to some instrument maker or optician than to enter a laboratory. Yet for those who, with these qualifications, decide to take the step, so initially alluring in its high wages, short hours, and easy work, some arrangement should be made to save them from the rut which the present unsatisfactory state of affairs leads to.

The matter is one hospital workers may with advantage to themselves and the community take into consideration, with a view to finding some means whereby the knowledge and skill acquired during these two years of laboratory work may be profitably and economically utilised. In the meantime the above suggestion may serve as a basis for discussion and criticism.

